# Impact of an antimicrobial stewardship-based infection prevention bundle on postoperative central nervous system infection in a tertiary neurosurgery department: a quasi-experimental study

**DOI:** 10.3389/fphar.2026.1856300

**Published:** 2026-08-27

**Authors:** Yuan Zhang, Binge Chang, Huanyu Wang, Yi Zhang, Haixia Ren

**Affiliations:** 1 Department of Pharmacy, Tianjin First Central Hospital, Tianjin, China; 2 Department of Neurosurgery, Tianjin First Central Hospital, Tianjin, China

**Keywords:** antibiotic use density, antimicrobial stewardship, defined daily doses, infection prevention bundle, postoperative central nervous system infection

## Abstract

**Background:**

Antimicrobial stewardship integration into infection prevention bundles remains inadequately addressed in neurosurgery.

**Aim:**

To evaluate the impact of an antimicrobial stewardship-based infection prevention bundle on postoperative central nervous system infection (PCNSI) incidence, antibiotic use density (AUD), and consumption of vancomycin and meropenem.

**Methods:**

A quasi-experimental before-and-after study compared 6-month periods before and after implementation. The bundle comprised perioperative optimization, infection control enhancements, surgical site and drainage management, microbiological stewardship, and antimicrobial stewardship components.

**Results:**

A total of 1,535 patients were analyzed (782 pre-intervention period, 753 post-intervention period). Surgical case-mix shifted significantly, with increased neurotrauma (15.73%–23.37%) and decreased cerebrovascular (45.40%–39.84%) and CSF/hydrocephalus (6.14%–2.79%) procedures. Overall PCNSI incidence decreased from 2.81% to 1.86% [0.654 (95% CI: 0.332–1.289); *P* = 0.217]. Neurotrauma‐specific PCNSI declined from 5.69% to 1.14% [0.190 (95% CI: 0.039–0.933); unadjusted *P* = 0.035], but it did not remain significant after correction for multiple comparisons. Monthly AUD decreased by 12.0% (*P* = 0.022). Vancomycin and meropenem consumption decreased by approximately 55% across all metrics (all *P* < 0.05), with large to very large effect sizes (Cohen’s d = 1.561–2.604).

**Conclusion:**

The bundle was associated with significant reductions in special‐use antimicrobial consumption, supporting practical implications for resistance mitigation and safety. The overall PCNSI decrease was not statistically significant. These findings are consistent with potential intervention benefit, pending validation in adequately powered prospective studies.

## 1 Introduction

Neurosurgical procedures are associated with a disproportionately high incidence of surgical site infections (SSIs) (Abdel-Latif et al., 2022). A systematic review and meta-analysis reported SSI rates of 4.03% at one month and 6.17% at three months postoperatively (Lee et al., 2024). Of particular concern, neurosurgical SSIs can precipitate severe complications, including meningitis, encephalitis, epidural abscess, subdural empyema, brain abscess, bone-flap infection, and wound infection, collectively defined as postoperative central nervous system infection (PCNSI)—potentially life-threatening conditions that significantly compromise patient survival and quality of life while imposing substantial burdens on healthcare resources (Venkatapura et al., 2021; Hussein et al., 2017; Mostafa et al., 2022; Shetty et al., 2017).

The incidence of PCNSI following intracranial procedures varies considerably (1%–8%) (McClelland and Hall, 2007; Korinek et al., 2006; National nosocomial infection surveillance [NNIS)]National Noscomial Infection Surveillance System, 2003; Chang et al., 2003; Vernet et al., 2004; Erman et al., 2005), with large-scale studies documenting rates of 5%–7%, escalating to approximately 10% without antibiotic prophylaxis (McClelland and Hall, 2007; Korinek et al., 2006). While perioperative antimicrobial prophylaxis effectively reduces PCNSI incidence (Ahmed et al., 2022), inappropriate practices—including prolonged duration, empiric broad-spectrum use, and suboptimal timing—may promote antimicrobial resistance and escalate healthcare costs (Ahmed et al., 2022; Harbarth et al., 2000). These concerns have prompted the development of national guidelines to standardize prophylactic protocols (Bratzler and Houck, 2004). However, despite advances in infection control measures, complete elimination of postoperative infections remains elusive (Buang and Haspani, 2012).

Optimizing antimicrobial utilization and infection control in surgical settings has emerged as an increasingly urgent priority (Goncalve et al., 2025). Antimicrobial stewardship programs (ASPs), though integral to infectious disease management, remain inadequately integrated into surgical practice (Dellit et al., 2007). Perioperative infection prevention bundles (IPBs)-comprising evidence-based interventions such as appropriate surgical antimicrobial prophylaxis (SAP), intraoperative euglycemia, and normothermia-have demonstrated efficacy in reducing SSIs across multiple specialties, including neurosurgery (Rubeli et al., 2019; Ma et al., 2017; Liu et al., 2017).

Multimodal antimicrobial stewardship-based infection prevention bundles, which integrate stewardship components with perioperative optimization and infection control measures, have shown promise in improving process outcomes and antimicrobial utilization (Balagna et al., 2023). However, such bundle remains limited in Chinese hospitals, with scarce published accounts of successful experiences (Xin et al., 2025).

This study aims to implement such a bundle in the neurosurgery department and evaluate its impact on PCNSI incidence, AUD, and consumption of vancomycin and meropenem. We use this term ‘bundle’ to reflect the integrated, multifaceted nature of the intervention, while recognizing that antimicrobial stewardship components provided its organizational framework.

## 2 Materials and methods

### 2.1 Study design

This single-center, quasi-experimental study compared PCNSI incidence, AUD, and consumption of vancomycin and meropenem among neurosurgical inpatients before and after implementation of an antimicrobial stewardship-based infection prevention bundle at Tianjin First Central Hospital. The pre-intervention period spanned July to December 2024, while the post-intervention period encompassed July to December 2025.

### 2.2 Setting and population

Tianjin First Central Hospital is a tertiary general hospital in Tianjin, China, with a 150-bed neurosurgery service comprising general wards and a dedicated neurosurgical intensive care unit (NICU). The department manages a broad spectrum of conditions, including neurotrauma, cerebrovascular disease, functional neurosurgery, and so on.

Analysis of AUD data from July to December 2024 revealed that elevated consumption was driven predominantly by extensive utilization of special-use antibiotics—specifically vancomycin and meropenem-prescribed almost exclusively for documented or suspected intracranial infections. Consequently, reducing AUD and curbing special-use antibiotics consumption was contingent upon lowering PCNSI incidence.

This study was approved by the Institutional Review Board of Tianjin First Central Hospital and conducted in accordance with the Declaration of Helsinki and national ethical guidelines. Written informed consent was obtained from all patients or their legally authorized representatives for the use of their medical records for research purposes. All data were extracted from the Hospital Information System (HIS).

### 2.3 Participants

Inclusion criteria were as follows: (1) patients aged 18–80 years who underwent neurosurgical procedures during the study period; (2) patients or their legally authorized representatives provided written informed consent; and (3) patients were able to receive 90-day postoperative follow-up, during which all patients were systematically monitored for PCNSI incidence regardless of clinical presentation.

Exclusion criteria were as follows: (1) patients who did not undergo craniotomy; (2) patients with external ventricular drainage; (3) patients with a history of craniotomy within three months; (4) patients with immunodeficiency disorders or severe chronic comorbidities (cardiac, pulmonary, renal, or hepatic dysfunction); (5) patients with pre-existing intracranial infection prior to index surgery; (6) patients with subarachnoid space adhesion causing CSF circulation impairment; (7) patients who declined participation before surgery; (8) patients whose primary neurosurgical procedure was performed at another institution.

### 2.4 Definitions

Surgical site infections (SSIs) were defined according to the Centers for Disease Control and Prevention (CDC) criteria (Centers for Disease Control and Prevention, 2017). The surveillance period for procedure-specific: 30 days for superficial and deep incisional infections following carotid endarterectomy and laminectomy, and 90 days for organ/space infections following craniotomy, spinal fusion, and ventricular shunt placement. Incisional SSIs were classified as superficial (involving skin and subcutaneous tissue), deep (involving fascia and/or muscle layers), or organ/space (involving osteomyelitis, disc space, intracranial structures, brain abscess, dura, meninges, ventriculitis, spinal abscess without meningitis, or vascular structures).

Postoperative central nervous system infection (PCNSI) was defined as a composite outcome encompassing meningitis, encephalitis, epidural abscess, subdural empyema, brain abscess, bone-flap infection, and/or wound infection (Stienen et al., 2019).

Diagnosis criteria of PCNSI were established based on the 2017 Infectious Disease Society of America (IDSA) clinical practice guidelines for healthcare-associated ventriculitis and meningitis (Tunkel et al., 2017). Definite PCNSI required fulfillment of clinical criteria plus microbiological confirmation:

Clinical criteria (≥1 required): (1) Body temperature > 38 °C without alternative infectious source, with or without new-onset headache, meningeal irritation, seizures, or altered mental status; (2) Computed tomography (CT) or magnetic resonance imaging (MRI) demonstrating diffuse cerebral edema, dural enhancement, or ventricular dilatation; (3) Peripheral leukocytosis (> 10 × 10^9^/L) or neutrophil predominance (> 80%); (4) Cerebrospinal fluid (CSF) analysis showing: ① Lumbar puncture: CSF pressure > 200 mmH_2_O); ② General characteristics of CSF: CSF yellow, cloudy, or purulent; ③ Leukocyte count in CSF > 500 × 10^6^/L with polymorphonuclear predominance (> 80%); ④ CSF biochemistry: glucose < 2.8 mmoL/L and protein > 0.45 g/L.

Microbiological confirmation (required for definite diagnosis): positive CSF culture, or pathogen detection by CSF metagenomic next-generation sequencing (mNGS).

Clinical suspicion of PCNSI following neurosurgery required fulfillment of at least one of the following two criteria: (1) Body temperature > 38 °C without identifiable alternative infectious source, accompanied by new-onset headache, meningeal irritation, seizures, or altered mental status; and/or (2) Peripheral leukocytosis (>10 × 10^9^/L) or neutrophil predominance (> 80%).

Antibiotic use density (AUD) was expressed as defined daily doses per 100 patient-days (DDDs/100 PD), and was calculated as (total DDDs ÷ total patient-days) × 100. The national compliance threshold for tertiary general hospitals in China is ≤ 40 DDDs/100 PD. For this study, a department-specific target of <35 DDDs/100 PD was established by the hospital antimicrobial stewardship committee in collaboration with the neurosurgery department, based on retrospective analysis of the department’s three-year antimicrobial utilization trends, disease case-mix profiles, and structured prescribing appropriateness reviews. This target is dynamic and subject to periodic reassessment.

Defined daily dose (DDD), established by the World Health Organization (WHO) Collaborating Centre for Drug Statistics Methodology, represents the assumed average maintenance dose per day for a drug’s main indication in adults. DDDs were calculated by dividing total consumption (mg) by the WHO-assigned DDD value (mg / DDD) obtained from the official ATC / DDD 2026 index published in Oslo, Norway (WHO Collaborating Centre for Drug Statistics Methodology, 2026).

### 2.5 Identification of risk factors

Based on antimicrobial utilization data from the pre-intervention period (July-December 2024), the multidisciplinary expert group conducted a comprehensive analysis of factors contributing to elevated PCNSI incidence and AUD. The following modifiable risk factors and practice deficiencies were identified: (1) suboptimal preoperative hair removal technique and timing; (2) inadequate perioperative glycemic control in diabetic patients; (3) preoperative malnutrition (body mass index < 18.5 kg/m^2^ or serum albumin < 30 g/L); (4) suboptimal intraoperative maintenance of normothermia and tissue oxygenation; (5) non-adherence to evidence-based surgical antimicrobial prophylaxis protocols; (6) deficient postoperative drainage tube management; (7) inconsistent aseptic technique and substandard hand hygiene compliance; (8) non-standardized CSF sampling protocols; (9) inappropriate antimicrobial selection (10) unjustified combination antimicrobial therapy; and (11) insufficient information technology infrastructure to support stewardship activities.

### 2.6 Antimicrobial stewardship-based infection prevention bundle implementation

It is important to clarify that the intervention evaluated in this study represents an antimicrobial stewardship-based infection prevention bundle, rather than a standalone ASP. While organized under the ASP governance framework, the intervention encompassed multidisciplinary activities extending beyond traditional stewardship boundaries, including perioperative care optimization (normothermia, glycemic control, drainage management), infection control enhancements (hand hygiene, barrier precautions, environmental cleaning), microbiological stewardship (standardized specimen collection, rapid diagnostic feedback), and antimicrobial stewardship components (prophylaxis guidelines, restricted drug approval, therapeutic monitoring). The observed outcomes therefore reflect the synergistic effects of this multifaceted approach. The bundle implementation team comprised neurosurgeons, infection control practitioners, anesthesiologists, clinical nurse specialists, infectious disease physicians, clinical pharmacists, and clinical microbiologists. The team convened biweekly meetings to review PCNSI cases, analyze antimicrobial utilization, provide recommendations, and establish implementation priorities. Specific improvement strategies are detailed in Table 1.

**TABLE 1:** Components of the antimicrobial stewardship-based infection prevention bundle implemented in the neurosurgery department.

Component	Measure
Neurosurgeons	1. Perioperative protocol adherence: Active implementation of standardized preoperative skin preparation, timely antimicrobial prophylaxis administration, and intraoperative normothermia and glycemic control protocols 2. Prescribing accountability: Strict adherence to bundle-defined surgical antimicrobial prophylaxis guidelines, including agent selection, dosing, timing, and duration, with prospective audit and feedback by clinical pharmacists 3. Emergency neurotrauma optimization: Leading the implementation of newly established emergency craniotomy prophylaxis protocols, which historically lacked standardized antimicrobial coverage prior to the intervention 4. Participation in biweekly bundle team meetings: Collaborative review of PCNSI cases, antimicrobial utilization data, and prescriber feedback reports to inform continuous quality improvement
Anesthesiologist	1. Prolonged procedure antimicrobial redosing: For surgical procedures exceeding 3 h in duration, an automated alert system for intraoperative antimicrobial redosing was developed in collaboration with the information technology department and integrated into the electronic health record system 2. Intraoperative physiological optimization: Enhanced perioperative maintenance of normothermia (core temperature ≥ 36 °C), adequate tissue oxygenation (peripheral oxygen saturation [SpO_2_] ≥ 95%), and optimal fluid management to ensure sufficient tissue perfusion pressure
Clinical pharmacists	1. Comprehensive medication management oversight: Pharmacists monitored multiple dimensions of antimicrobial therapy, including: (i) implementation of individualized medication plans; (ii) therapeutic efficacy assessment; (iii) patient adherence evaluation; (iv) pharmacogenomic testing and interpretation; and (v) therapeutic drug monitoring (TDM) 2. Guideline development and dissemination: A standardized neurosurgical antimicrobial prophylaxis guideline was developed and formally announced to all clinical staff. Subsequently, clinical pharmacists conducted a series of educational sessions on antimicrobial stewardship principles and evidence-based surgical prophylaxis. Monthly prescribing pattern audits were performed to evaluate improvement in antimicrobial utilization, specifically assessing: appropriateness of agent selection, route of administration, timing of prophylactic dosing, dosage regimens, and prophylaxis duration. Audit findings were communicated to surgeons through structured feedback reports 3. Special-use antimicrobial stewardship: Stringent oversight of the approval process for special-use antibiotics in the neurosurgery department was implemented, with designated pharmacist authorization required for special-use antibiotics requests
Infectious disease physicians	1. Differentiation of infection versus colonization: Accurate distinction between true bacterial infection and microbial colonization, enabling evidence-based antimicrobial decision-making and avoidance of unnecessary antibiotic exposure 2. Dynamic therapeutic monitoring and optimization: Systematic surveillance of clinical and laboratory parameters, including cerebrospinal fluid analysis (cell count, protein, glucose), serum inflammatory markers [C-reactive protein (CRP), procalcitonin (PCT)], and clinical signs of infection. Based on integrated assessment of microbiological data, biomarker trends, and clinical status, infectious disease specialists provided timely recommendations for antimicrobial regimen modification, including agent selection, dosing optimization, and treatment de-escalation or discontinuation, to ensure optimal patient outcomes
Clinical microbiologists	1. Standardized specimen collection training: Clinical microbiologists provided systematic education to physicians and nursing staff regarding optimal techniques for culture specimen collection. This initiative aimed to ensure accurate and reliable pathogen identification from neurosurgical patients 2. CSF collection protocol optimization: CSF specimens were obtained via lumbar puncture rather than from indwelling drainage devices. Paired aerobic and anaerobic blood culture bottles were submitted simultaneously to maximize detection of fastidious organisms, including *Propionibacterium acnes*, which typically requires anaerobic culture conditions 3. Rapid diagnostic communication: Although CSF gram stain sensitivity is inferior to culture, positive smear results triggered immediate notification to the bundle team via secure communication platforms. Definitive culture and antimicrobial susceptibility results were communicated promptly upon availability. Real-time access to laboratory information system data was integrated into the Hospital information system (HIS) to facilitate expedited result retrieval by clinical teams
Clinical nurse specialists	1. Preoperative skin preparation: Execution of standardized preoperative hair removal and skin antisepsis protocols in accordance with institutional guidelines 2. Surgical drainage management: Maintenance of patent wound drainage systems, including: (i) Ensuring unobstructed flow; (ii) meticulous observation and documentation of drainage characteristics (volume, color, consistency); and (iii) strict adherence to aseptic technique during all manipulation procedures
Infection control practitioners	1. Protocol review and staff competency: Systematic evaluation and updating of infection prevention protocols, coupled with comprehensive training of healthcare personnel on: (i) Hand hygiene compliance; (ii) environmental cleaning and equipment disinfection; (iii) patient bathing and oral hygiene; and (iv) daily assessment of catheter, probe, and drain removal readiness 2. Surveillance and performance feedback: Monitoring of adherence rates through structured compliance checklists, with periodic disclosure of healthcare-associated infection (HAI) incidence via bundle team meetings to drive continuous quality improvement

Information technology systems were leveraged to establish tiered prescribing privileges and automated alerts within the Hospital Information System (HIS), with account restrictions configured according to antimicrobial classification and prescriber authorization levels. Standardized protocols for perioperative surgical antimicrobial prophylaxis were developed to prevent inappropriate utilization.

### 2.7 Follow-up duration and outcome indicators

Post-intervention cohort patients were followed for 90 days postoperatively through telephone interviews, outpatient clinic visits, and systematic electronic medical record review. Surveillance documented PCNSI occurrence, antimicrobial prescribing patterns, treatment duration, and microbiological culture results.

Patients who transferred to other hospitals, voluntarily withdrew from treatment, or were lost to follow-up during the 90-day surveillance period were recorded as incomplete follow-up. Their data were included in the analysis up to the point of censoring.

Primary outcomes: PCNSI incidence.

Secondary outcomes: monthly AUD, DDDs and monthly AUD of vancomycin and meropenem.

### 2.8 Statistical analysis

Statistical analyses were performed using SPSS version 26.0 (IBM Corp., Armonk, NY, Uinted States). Categorical variables are presented as frequencies and percentages. Continuous variables were presented as mean ± standard deviation (SD) or median (interquartile range, IQR) depending on the distribution, which was assessed using the Shapiro-Wilk test. Given the pre-and post intervention data were collected from distinct time periods (July-December 2024 vs. July-December 2025) with only six monthly observations per period, independent-sample t-tests or Mann-Whitney U tests were used for continuous metrics (monthly AUD, DDDs and monthly AUD of vancomycin and meropenem), and Pearson’s chi-squared tests or Fisher’s exact tests for categorical outcomes (PCNSI incidence, surgical type distribution). Effect sizes (Cohen’s d, r = Z/√N, and Cramer’s V/Phi) are reported to supplement *P*-values. All tests were two-tailed, and a *P*-value < 0.05 was considered statistically significant.

The standardized rate was calculated asΣ(P_ᵢ_^pre^ × R_ᵢ_^post^), where P_ᵢ_^pre^ represents the proportion of surgical type *i* in the pre-intervention period, and R_ᵢ_^post^ represents the observed PCNSI rate for surgical type *i* in the post-intervention period.

Post-hoc power analysis was conducted using G*Power 3.1.9.7 (Faul et al., 2009) based on the observed effect sizes and sample sizes to evaluate the statistical power of our primary comparisons.

## 3 Result

**FIGURE 1:**
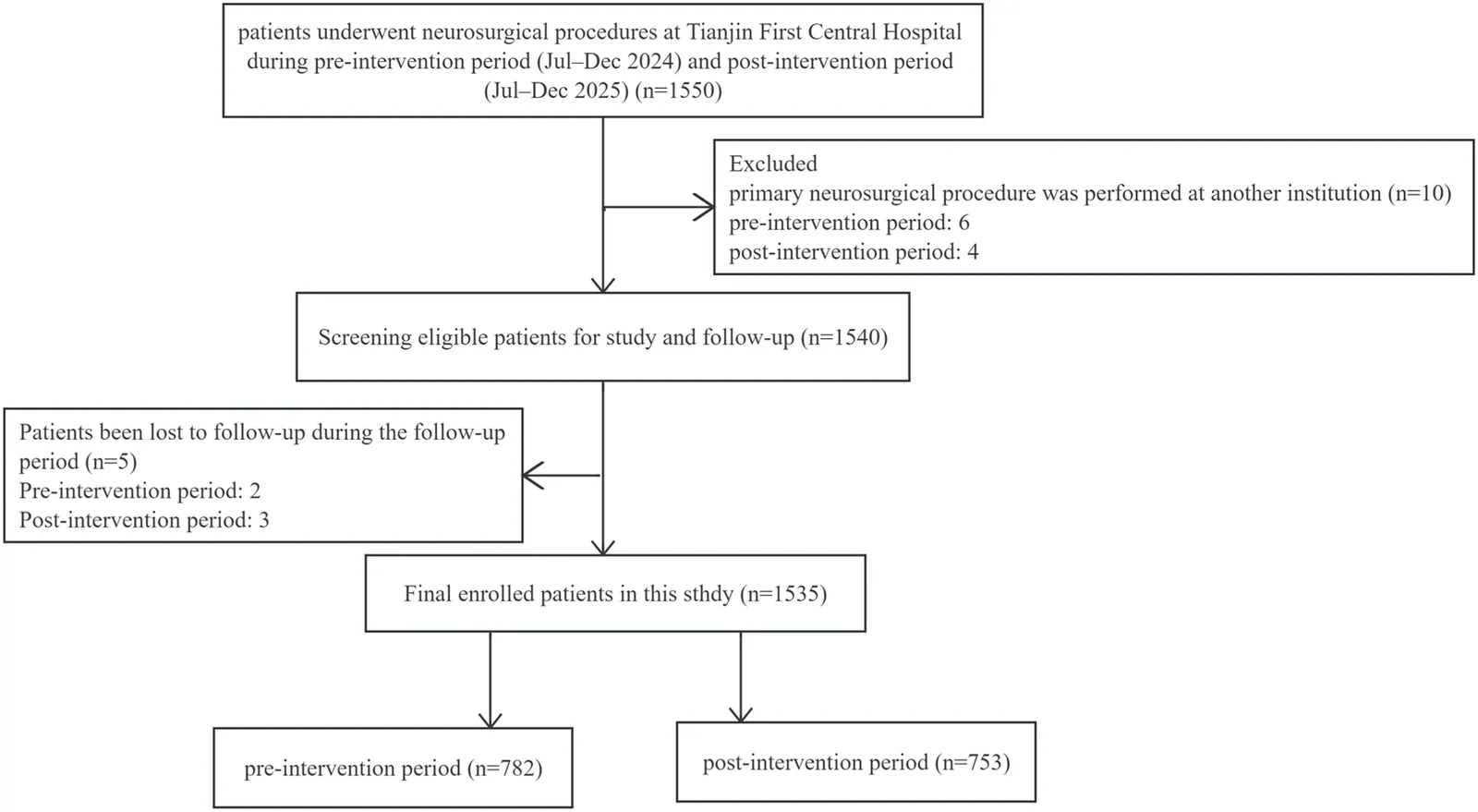
Consort flow diagram of patient enrollment and analysis in an antimicrobial stewardship-based infection prevention bundle study in neurosurgery. A total of 1,550 patients who underwent neurosurgical procedures at Tianjin First Central Hospital during July–December 2024 and July–December 2025 were screened. Exclusion criteria comprised primary surgery at another institution (n = 10) and loss to follow-up (n = 5). The final analytical cohort comprised 1,535 patients: 782 in the pre-intervention period and 753 in the post-intervention period.

A total of 1,535 neurosurgical procedures were included in the analysis: 782 in the pre-intervention period and 753 in the post-intervention period. Of 1,550 patients screened, 15 were excluded-10 due to primary surgery at another institution and 5 due to loss to follow-up (Figure 1). Procedures were categorized into eight types: neurotrauma, functional neurosurgery, spinal, cerebrospinal fluid (CSF)/hydrocephalus, cerebrovascular, neuro-oncology, peripheral nerve, and miscellaneous.

Comparison of surgical case mix revealed significant differences between periods. Neurotrauma surgeries were significantly less frequent in the pre-intervention period compared with the post-intervention period [123/782 (15.73%) versus 176/753 (23.37%); *χ* ^*2*^ = 14.292, *P* < 0.001]. Conversely, CSF/hydrocephalus procedures [48/782 (6.14%) versus 21/753 (2.79%); *χ* ^*2*^ = 10.024, *P* = 0.002] and cerebrovascular surgeries [355/782 (45.40%) versus 300/753 (39.84%); *χ* ^*2*^ = 4.840, *P* = 0.028] were significantly more frequent pre-intervention. No statistically significant differences were observed in the distribution of other surgical categories (Table 2).

**TABLE 2:** Characteristics of surgical procedures performed.

Type of surgery	Number of surgeries in pre-intervention period n (%)	Number of surgeries in post-intervention period n (%)	Standardized residuals	Individual χ^2^	*p*
Neurotrauma	123 (15.73)	176 (23.37)^a^	−2.4/2.4	14.292	<0.001
Functional neurosurgery	76 (9.72)	74 (9.83)	0.0/0.0	0.005	0.943
Spinal	21 (2.69)	15 (1.99)	0.6/−0.6	0.805	0.370
CSF/Hydrocephalus	48 (6.14)	21 (2.79)^b^	2.2/−2.2	10.024	0.002
Cerebrovascular	355 (45.40)	300 (39.84)^c^	1.2/−1.2	4.840	0.028
Neuro-oncology	87 (11.13)	107 (14.21)	−1.2/1.2	3.306	0.069
Peripheral nerve	12 (1.54)	19 (2.52)	−1.0/1.0	1.895	0.169
miscellaneous	60 (7.67)	41 (5.44)	1.2/−1.2	3.097	0.078
Total	782	753	​	​	​
Overall χ^2^	​	32.285			
p	​	<0.001			

Values are presented as n (%). a,b,c Individual 2 × 2 chi-squared comparisons for categories with standardized residuals > |2.0|. These pairwise comparisons are exploratory and were not adjusted for multiple testing. Overall test: Pearson’s chi-squared test for 7 × 2 contingency table.

During the pre-intervention period, 22 PCNSIs occurred (2.81%, 22/782). By surgical type: neurotrauma (7), cerebrovascular (7), functional neurosurgery (4), neuro-oncology (3), and CSF/hydrocephalus (1). During the post-intervention period, 14 PCNSIs occurred (1.86%, 14/753). by type: functional neurosurgery (5), cerebrovascular (5), neurotrauma (2), neuro-oncology (1), and CSF/hydrocephalus (1) (Table 3).

**TABLE 3:** Comparison of PCNSI incidence by surgical type: pre-versus post-intervention periods.

Type of surgery	PCNSI in pre-intervention period	​	PCNSI in post-intervention period	​	*F*- statistic	*P*-value
	Number of PCNSI/total number of surgeries	PCNSI incidence (%)	Number of PCNSI/total number of surgeries	PCNSI incidence (%)	*F*	*P*
Neurotrauma	7/123	5.69	2/176^b^	1.14	5.145	0.035
Functional neurosurgery	4/76	5.26	5/74	6.76	​	​
Spinal surgery	0/21	0	0/15	0	​	​
CSF/Hydrocephalus	1/48	2.08	1/21	4.76	​	​
Cerebrovascular	7/355	1.97	5/300	1.67	​	​
Neuro-oncology	3/87	3.45	1/107	0.93	​	​
Peripheral nerve	0/12	​	0/19	0	​	​
miscellaneous	0/60	​	0/41	0	​	​
Total	22/782	2.81	14/753	1.86	​	​
*F*	2.571				​	​
*p*	0.897				​	​

Values are presented as n (%). All *P*-values are calculated using Pearson’s Chi-square or Fisher’s exact test as appropriate.PCNSI, postoperative central nervous system infection.

Monthly PCNSI trends were descriptively monitored (Table 4). Overall PCNSI incidence decreased from 2.81% (22/782) to 1.86% (14/753) [0.654, (95% CI: 0.332–1.289; *P* = 0.217)]. The 95% CI lower bound (RR 0.332) corresponds to a 67% relative risk reduction. Post-hoc power analysis revealed that this study had 24% power to detect the observed effect size (Cramer’s V = 0.032, χ^2^ = 1.525, *N* = 1,535).

**TABLE 4:** Monthly PCNSI incidence: comparison of pre-versus post-intervention periods.

Months	PCNSI in pre-intervention period	​	PCNSI in post-intervention period	​	*F*-statistic	*P*-value
	Number of PCNSI/total number of surgeries	PCNSI incidence	Number of PCNSI/total number of surgeries	PCNSI incidence	*F*	*P*
July	3/112	2.68	3/150	2	1.525	0.217
August	5/133	3.76	2/140	1.43		
September	2/133	1.5	2/148	1.35		
October	3/161	1.87	3/138	2.17		
November	4/154	2.6	2/92	2.17		
December	5/89	5.62	2/85	2.35		
Total	22/782	2.81	14/753	1.86	​	​
*F*	1.536				​	​
*P*	0.597				​	​

Values are presented as n (%). All *P*-values are calculated using Pearson’s Chi-square or Fisher’s exact test as appropriate. PCNSI, postoperative central nervous system infection.

The neurotrauma subgroup showed a numerical reduction [7/ 123 (5.69%–2/176 (1.14%)], with a relative risk of 0.19 (95% CI: 0.039–0.933; unadjusted *P* = 0.035) indicating an 80% relative risk reduction with a confidence interval that excludes the null value. However, this did not remain significant after Bonferroni correction (α’ = 0.007; corrected *P* = 0.245).

The observed shift in surgical case mix—with a 43% relative increase in high-risk neurotrauma procedures [176/782 (23.37%) versus 123/753 (15.73%); *χ* ^*2*^ = 14.292, *P* < 0.001] and significant decreases in CSF/hydrocephalus [48/782 (6.14%) versus 21/753 (2.79%); *χ* ^*2*^ = 10.024, *P* = 0.002] and cerebrovascular [355/782 (45.40%) versus 300/753 (39.84%); *χ* ^*2*^ = 4.840, *P* = 0.028] procedures—introduced potential for confounding. To assess whether these case-mix differences confounded the observed PCNSI incidence reduction, we performed direct standardization of the post-intervention PCNSI rate using the pre-intervention surgical distribution as the reference population. The directly standardized PCNSI incidence for the post-intervention period was 1.99%, marginally higher than the crude observed rate of 1.86% (14/753). To further validate this finding, we performed the converse analysis: applying the post-intervention case mix to pre-intervention type-specific rates yielded a standardized pre-intervention rate of 3.18% (vs. observed 2.81%).

Implementation of the antimicrobial stewardship-based infection prevention bundle was associated with a significant 11.97% reduction in monthly AUD, from 39.02 ± 2.47 to 34.35 ± 3.44 (t = 2.704, *P* = 0.022), approaching the institutional target of < 35 DDDs/100 PD (Table 5).

**TABLE 5:** Monthly AUD: comparison of pre-versus post-intervention periods.

Month	In pre-intervention period	In post-intervention period
	AUD (DDDs/100 PD)	AUD (DDDs/100 PD)
Jul	41.97	33.17
Aug	40.78	37.34
Sep	38.06	37.19
Oct	38.78	37.47
Nov	34.86	29.86
Dec	39.69	31.06
Mean + SD	39.02 ± 2.47	34.35 ± 3.44
*t*	​	2.704
*P*	​	0.022

Values are presented as median ± SD. *P*-value is calculated using independent-sample t-tests. AUD, antimicrobial use density.

Vancomycin consumption decreased significantly by 57.69% in DDDs (from 104.41 ± 28.89 to 44.18 ± 15.33; t = 4.511, *P* = 0.001) and by 52.9% in AUD (from 2.59 ± 0.58 to 1.22 ± 0.47; t = 4.495, P = 0.001). Similarly, meropenem consumption decreased by 54.82% in DDDs (from 177.75 ± 67.37 to 80.31 ± 21.78; t = 3.371, P = 0.007) and by 54.32% in AUD (from 4.75 + 1.88 to 2.17 ± 0.53; t = 3.229, P = 0.009) (Table 6). All antibiotic consumption metrics declined significantly with large effect sizes: monthly AUD (Cohen’s d = 1.561), vancomycin DDDs (Cohen’s d = 2.604), vancomycin AUD (Cohen’s d = 2.595), meropenem DDDs (Cohen’s d = 1.946) and meropenem AUD (Cohen’s d = 1.864) (all *P* < 0.05) (Table 7).

**TABLE 6:** Consumption of vancomycin and meropenem: comparison of pre-versus post-intervention periods.

​	In pre-intervention period				In post-intervention period			
Month	Vancomycin (DDDs)	Vancomycin (AUD)	Meropenem (DDDs)	Meropenem (AUD)	Vancomycin (DDDs)	Vancomycin (AUD)	Meropenem(DDDs)	Meropenem (AUD)
Jul	117	3.25	273.49	7.59	49.1	1.43	67.83	1.98
Aug	139.3	2.53	179.3	4.54	53	1.43	62.66	1.69
Sep	128	3.29	225.84	5.8	56.5	1.50	91.34	2.43
Oct	63.25	1.81	179.17	5.12	57	1.70	91.67	2.73
Nov	82.4	2.23	89.67	2.43	24	0.63	55.67	1.47
Dec	96.5	2.44	119	3.01	25.5	0.61	112.68	2.70
Mean + SD	104.41 ± 28.89	2.59 ± 0.58	177.75 ± 67.37	4.75 ± 1.88	44.18 ± 15.33	1.22 ± 0.47	80.31 ± 21.78	2.17 ± 0.53
t	​	​	​	​	4.511	4.495	3.371	3.229
P	​	​	​	​	0.001	0.001	0.007	0.009

Values are presented as median ± SD. *P*-values are calculated using independent-sample t-tests. AUD, antimicrobial use density; DDDs, defined daily doses.

**TABLE 7:** Primary outcomes of pre- versus post-intervention periods.

Outcome	Pre-intervention period	Post-intervention period	P	Effect size	RR/MD (95% CI)
PCNSI, %	2.81	1.86	0.217	0.032	0.654 (0.332–1.289)
Neurotrauma PCNSI, %	5.69	1.14	0.035	0.131	0.190 (0.039–0.933)
Monthly AUD	39.02 ± 2.47	34.35 ± 3.44	0.022	1.561	4.67(0.823–8.527)
Vancomycin DDDs	104.41 ± 28.89	44.18 ± 15.33	0.001	2.604	60.23 (30.476–89.974)
Vancomycin AUD	2.59 ± 0.58	1.22 ± 0.47	0.001	2.595	1.37 (0.694–2.050)
Meropenm DDDs	177.75 ± 67.37	80.31 ± 21.78	0.007	1.946	97.44 (33.031–161.843)
Meropenrm AUD	4.75 ± 1.88	2.17 ± 0.53	0.009	1.864	2.58 (0.800–4.363)

Values are mean ± SD, or %. PCNSI, postoperative central nervous system infection; AUD, antimicrobial use density; DDDs, defined daily doses; RR, relative risk; MD, mean difference. Effect sizes: Cohen’s d (t-tests), r = Z/√N (Mann-Whitney U), Phi (χ^2^).

## 4 Discussion

Our neurosurgery service comprises two units with 150 beds in total, including general wards and a dedicated neurosurgical intensive care unit (NICU). The department performs a diverse range of neurosurgical procedures, including neurotrauma, cerebrovascular, functional neurosurgery, and so on. The NICU admits postoperative neurosurgical patients, the majority of whom have experienced significant intraoperative blood loss requiring transfusion and remain unconscious for extended periods (Sachdeva et al., 2017). These factors are recognized as independent risk factors for healthcare-associated infections. Postoperative meningitis, in particular, represents a major complication following neurosurgery (Iwasaki et al., 2017), resulting in prolonged hospitalization and increased antimicrobial exposure that exerts selective pressure for multidrug-resistant organisms (MDROs). Moreover, our NICU is staffed by neurosurgeons who may have limited expertise in postoperative critical care management and antimicrobial therapy. Therefore, implementation of an antimicrobial stewardship-based infection prevention bundle in this setting is warranted, particularly given the limited published evidence on antimicrobial stewardship in neurosurgical populations. Antimicrobial stewardship programs constitute a critical element of clinical quality governance, with AUD serving as the primary metric for evaluating antimicrobial utilization and prescribing appropriateness (Durand et al., 2024). This metric also serves as a key performance benchmark in the national assessment of tertiary public hospitals in China.

This study evaluated the impact of an antimicrobial stewardship-based infection prevention bundle on perioperative care, infection control practices, microbiological stewardship, and antimicrobial utilization in the neurosurgery department. Following 6 months of implementation, the overall PCNSI incidence demonstrated a numerical reduction from 2.81% to 1.86% [0.654, (95% CI: 0.332–1.289, *P* = 0.217)] that did not reach statistical significance. The point estimate suggests a clinically meaningful 34% relative risk reduction. Post-hoc power analysis indicated 24% power for the overall PCNSI comparison based on the observed effect size (Cramer’s V = 0.032, χ^2^ = 1.525, N = 1535), suggesting limited ability to detect a difference of this magnitude. This low power reflects the small effect size (absolute difference: 2.81% vs. 1.86%) rather than inadequate sample size per se; indeed, approximately 7,664 patients would be required to achieve 80% power for an effect of this size. Consequently, the non-significant primary outcome should be interpreted as inconclusive due to limited statistical power, rather than as evidence of no intervention effect. However, the significant reductions observed across all antibiotic consumption metrics (all Cohen’s d > 1.5, *P* < 0.05) support the clinical effectiveness of the intervention.

Direct standardization demonstrated that applying the pre-intervention surgical distribution to post-intervention type-specific infection rates yielded a standardized rate of 1.99%, only marginally higher than the crude observed 1.86%. The direction of this difference is counter-intuitive: the increased proportion of neurotrauma procedures, which carry the highest intrinsic PCNSI risk, would theoretically elevate rather than reduce the overall post-intervention infection incidence. The fact that the standardized rate exceeds the observed rate indicates that case-mix differences attenuated the observed reduction. Conversely, applying the post-intervention case-mix to pre-intervention rates yielded 3.18%, substantially higher than the observed pre-intervention 2.81%. This confirms that baseline infection incidence would have been even higher if the pre-intervention period contained the same high-risk case-mix. Consequently, the observed reduction to 1.86% reflects genuine bundle effectiveness.

Neurotrauma procedures—a high-risk category for PCNSI—were significantly more frequent in the post-intervention period [176 (23.37%) versus 123 (15.73%); *χ* ^*2*^ = 14.292, *P* < 0.001]. Despite this unfavorable case-mix shift, neurotrauma-specific PCNSI incidence declined significantly [7 (5.69%) versus 2 (1.14%); χ^2^ = 5.145, unadjusted *P* = 0.035], representing an 80% relative risk reduction. This finding is biologically plausible, as emergency neurotrauma historically lacked standardized perioperative prophylaxis, which the bundle directly addressed. However, this subgroup analysis did not remain significant after Bonferroni correction (corrected *P* = 0.245) for multiple comparisons and should be interpreted as hypothesis-generating. Validation in adequately powered studies with prespecified subgroup hypotheses is warranted. This finding is consistent with prior ASP studies demonstrating improved process measures and antibiotic consumption without significant SSI reductions (Van Kasteren et al., 2005; Manniën et al., 2006; Kilani et al., 2017).

Prior to implementation, emergency neurotrauma patients presented a critical care gap: no standardized preoperative or intraoperative antimicrobial prophylaxis was administered. The bundle addressed this vulnerability through three core components: (i) evidence-based prophylaxis guidelines for emergency craniocerebral surgery; (ii) targeted education and competency assessment for emergency neurosurgical teams; and (iii) reliable supply chain protocols for prophylactic agents. Post-intervention audit demonstrated universal compliance with these protocols. The bundle additionally encompassed monthly prescribing audits with feedback. These measures collectively reduced antimicrobial consumption and PCNSI incidence, supporting the value of structured perioperative stewardship in neurosurgical practice.

Monthly AUD, from 39.02 ± 2.47 to 34.35 ± 3.44 (t = 2.704, *P* = 0.022), approaching the institutional target of < 35 DDDs/100 PD. Temporal analysis revealed that monthly AUD peaked at 41.97 DDDs/100 PD in July 2024, substantially exceeding this threshold. During four of six pre-intervention months (August-October and December 2024), AUD remained consistently above target. Following bundle implementation in July 2025, antimicrobial utilization declined progressively, falling below target in three of six post-intervention months (July, November, and December 2025), with a nadir of 29.86 DDDs/100 PD in November 2025.

According to international guidelines for intracranial infections (Schneider et al., 2022), vancomycin and/or meropenem are recommended as first-line empiric therapy. The Infectious Diseases Society of America (IDSA) and the European Society of Clinical Microbiology and Infectious Diseases (ESCMID) endorse vancomycin for bacterial meningitis, with meropenem or alternative β-lactams employed as adjunctive therapy based on local susceptibility patterns (Tunkel et al., 2017; ven de beek et al., 2016). German guidelines for community-acquired acute bacterial meningitis similarly recommend vancomycin combined with meropenem or ceftazidime, with metronidazole added when anaerobic coverage is indicated (Klein et al., 2023). Given the central role of these agents in neurosurgical infection management, this study evaluated bundle effectiveness through comparative analysis of vancomycin and meropenem consumption metrics.

The antimicrobial stewardship-based infection prevention bundle was associated with marked reductions in special-use antibiotic consumption. Vancomycin and meropenem consumption decreased by approximately 50% across all metrics (DDDs and AUD), with large to very large effect sizes (Cohen’s d = 1.864–2.604, all *P* < 0.05). These consistent reductions, irrespective of measurement methodology, suggest that the bundle effectively curtailed excessive empiric use of these agents. This finding is particularly relevant given that vancomycin and meropenem represent first-line empiric therapy for intracranial infections per international guidelines, yet their unrestricted use promotes nephrotoxicity and selective pressure for multidrug-resistant organisms. Notably, the magnitude of these reductions exceeds those reported in comparable stewardship interventions. A 6-month bundle at a Chinese tertiary hospital demonstrated a 30.1% reduction in total DDDs (*P* < 0.05) (Xin et al., 2025), While a 12-month implementation in an Italian vascular surgery unit achieved significant carbapenem reduction (mean AUD: 4.53 to 1.51; P = 0.01) (Vecchia et al., 2023).

An important contributor to the reduction in special-use antibiotics consumption was the implementation of specialized approval oversight. Pre-intervention, department heads held unrestricted prescribing authority for special-use antibiotics, with attending physicians frequently accessing departmental accounts for approval without individualized clinical assessment. Post-intervention, this process was centralized under dedicated infectious diseases clinical pharmacists, who assumed responsibility for vancomycin and meropenem authorization. This transition enabled rigorous indication assessment, real-time protocol adherence monitoring, and prospective audit with feedback to prescribers.

Several limitations of this study should be acknowledged. First, the single-centre design limits generalizability. Nevertheless, quasi-experimental before-and-after designs are feasible as pilot investigations for future randomized trials, and prior evidence demonstrates that even targeted educational interventions can optimize antimicrobial utilization. Second, post-hoc power analysis revealed that achieved power to detect the observed absolute reduction of 0.95 percentage points in overall PCNSI incidence [0.654, (95% CI: 0.332–1.289); *P* = 0.217] was approximately 24%. The non-significant primary outcome should therefore be interpreted as inconclusive rather than evidence of no effect. A sample size of approximately 7,664 patients (3,832 per group) would be required to achieve 80% power for this effect size. Third, baseline imbalances in surgical case mix between periods may have confounded PCNSI incidence comparisons. The post-intervention period included a significantly higher proportion of neurotrauma procedures, which carry intrinsically elevated infection risk. Although stratified analyses demonstrated significant reductions in neurotrauma-specific PCNSI incidence, residual confounding from unmeasured patient-level factors (e.g., injury severity scores, Glasgow Coma Scale at admission) cannot be excluded. Multivariable regression adjustment was precluded by the limited number of infection events. Fourth, the 6-month intervention period may be insufficient to capture delayed effects of stewardship interventions on infection outcomes. ASP-associated improvements in antimicrobial consumption and prescribing appropriateness may precede demonstrable reductions in PCNSI incidence by several months, as suggested by the progressive AUD decline observed in our temporal analysis. Longer follow-up periods are warranted to assess sustained effectiveness. Fifth, resource-specific contextual factors—including institutional funding, administrative support, dedicated infectious disease pharmacy expertise, and information technology infrastructure—may limit direct transferability of our intervention to resource-constrained settings. Geographic heterogeneity in pathogen prevalence and antimicrobial resistance patterns further suggests that protocol adaptation to local epidemiology is essential (Wen et al., 2023). Sixth, the exclusive focus on a single surgical service within an academic tertiary center limits applicability to community hospitals or non-neurosurgical specialties. The presence of specialized neurosurgical intensive care and onsite infectious disease consultation may overestimate intervention effectiveness in settings without comparable resources. Seventh, post-discharge antimicrobial utilization and clinical outcomes were not systematically captured, potentially obscuring complete treatment courses and long-term sequelae for PCNSI patients. This data gap precludes assessment of total antimicrobial exposure and post-discharge infection recurrence. Eighth, as a bundled intervention, our ASP-based infection prevention bundle comprised multiple simultaneous components, including preoperative antimicrobial optimization, intraoperative aseptic technique reinforcement, postoperative infection surveillance, pharmacist-led antimicrobial stewardship rounds, multidisciplinary team education, and so on. Consequently, we are unable to determine which specific component(s) contributed most to the observed improvements in PCNSI incidence and antibiotic consumption. This is an inherent limitation of bundle interventions, where synergistic effects between components may exceed the sum of individual effects. Future research employing factorial designs or sequential implementation strategies would be needed to disentangle the relative contributions of each component. Ninth, an important unmeasured source of potential bias involves the post-intervention shift to lumbar-puncture-based CSF sampling (rather than drainage tube sampling) for microbiological diagnosis, as implemented by the clinical microbiology team. This protocol change was intended to improve diagnostic accuracy by reducing contamination risk and enhancing recovery of true pathogens, including *Propionibacterium acnes* in anaerobic cultures. However, this modification may have introduced detection bias with paradoxical effects on observed PCNSI incidence. Specifically, improved sampling sensitivity could theoretically increase the apparent yield of positive cultures, thereby inflating rather than deflating detected infections. Conversely, more rigorous specimen quality could reduce false-positive cultures from contaminated drainage samples, potentially decreasing artifactual diagnoses. The net direction of this bias is indeterminate and cannot be quantified retrospectively. Furthermore, the altered sampling source may have changed the case mix of detected infections (e.g., proportion of true meningitis versus catheter-associated colonization), complicating temporal comparisons of pathogen profiles and antimicrobial susceptibility patterns. This protocol change was not randomized or systematically evaluated as an independent intervention component, and its contribution to observed outcome trends remains confounded with other bundle elements. Finally, persistent knowledge gaps among clinicians regarding antimicrobial pharmacology and stewardship principles were observed during implementation, with empirical broad-spectrum prescribing continuing in certain complex contexts. This underscores the necessity of ongoing education, iterative protocol refinement, and sustained leadership engagement. The observed reductions in monthly AUD and special-use antibiotics consumption, despite these challenges, suggest that structural interventions (e.g., formulary restriction, prospective audit with feedback) may be more immediately impactful than knowledge-based strategies alone.

## 5 Conclusion

In conclusion, this study evaluated an antimicrobial stewardship-based infection prevention bundle in neurosurgical practice. The intervention was associated with significant reductions in antimicrobial consumption metrics, with large to very large effect sizes (Cohen’s d = 1.561–2.604, all *P* < 0.05). These findings have practical implications for mitigating selective pressure for multidrug-resistant organisms and reducing vancomycin-associated nephrotoxicity. The decrease in overall PCNSI incidence was not statistically significant (*P* = 0.217, post-hoc power = 24%), consistent with limited power and prior ASP studies demonstrating improved process measures without significant infection incidence reductions. Neurotrauma-specific PCNSI showed a promising signal (5.69%–1.14%, *P* = 0.035) but did not remain significant after correction for multiple comparisons. The direction of effect across outcomes is consistent with potential intervention benefit; however, the quasi-experimental design, modest sample size, and inability to isolate individual bundle components limit causal inference. These findings support the feasibility of implementing structured antimicrobial stewardship programs in neurosurgical settings and provide effect size estimates for future trial design. Validation in adequately powered, multicenter prospective studies with prespecified subgroup hypotheses is warranted.

## Data Availability

The original contributions presented in the study are included in the article/supplementary material, further inquiries can be directed to the corresponding author.
